# Cortical microstructural change linked to clinical recovery in subacute delayed encephalopathy after acute carbon monoxide poisoning: a longitudinal case report

**DOI:** 10.3389/ftox.2025.1701308

**Published:** 2025-12-05

**Authors:** Takehiro Tamura, Yuka Fujimoto, Hironobu Nakamura, Yuki Takahashi, Junya Fujino, Shunsuke Takagi, Hidehiko Takahashi, Genichi Sugihara

**Affiliations:** 1 Department of Psychiatry and Behavioral Sciences, Graduate School of Medical and Dental Sciences, Institute of Science Tokyo, Tokyo, Japan; 2 Center for Brain Integration Research, Institute of Biomedical Engineering, Institute of Science Tokyo, Tokyo, Japan

**Keywords:** carbon monoxide poisoning, delayed encephalopathy after acute carbon monoxide poisoning (DEACMP), cortical microstructure, myelin mapping, cognitive impairment, delayed encephalopathy

## Abstract

Delayed encephalopathy after acute carbon monoxide poisoning (DEACMP) causes disabling cognitive–behavioral symptoms. While cortical atrophy is recognized as a correlate of long-term outcome, links between intracortical microstructural dynamics and clinical presentation remain largely unexplored. We followed a 51-year-old man with new-onset DEACMP for 5 months, using serial MRI combining cortical thickness with T1-weighted/T2-weighted (T1w/T2w) mapping as a proxy for intracortical microstructure. Despite progressive cortical thinning, T1w/T2w signals showed heterogeneous, region-specific trajectories. In the frontal networks, increases within premotor and dorsolateral prefrontal cortices aligned with improvements in executive function, whereas limited change in orbitofrontal/ventrolateral cortices was consistent with behavioral disinhibition. Overall, the clinical picture tracked more closely with intracortical signals than with morphometric atrophy. By combining T1w/T2w mapping with morphometry, this case provides the first longitudinal evidence of divergent cortical trajectories in subacute DEACMP—progressive thinning versus early intracortical improvement; confirmation in larger cohorts is warranted.

## Introduction

The syndrome of delayed encephalopathy after acute carbon monoxide poisoning (DEACMP) is characterized by the delayed onset of neuropsychiatric symptoms, which emerge days to weeks after an apparent recovery from acute poisoning (Wang et al., 2025; Huang et al., 2020). The transient asymptomatic period is typically called the lucid interval. After that interval, patients can develop a broad spectrum of cognitive and behavioral disturbances, most notably deficits in memory, attention, and executive functioning, which can persist and significantly impair social reintegration (Wang et al., 2025). The pathophysiology of DEACMP has not been fully elucidated; however, diffuse white matter injury—particularly demyelination—has consistently been implicated as a central mechanism (Beppu, 2014). Given that myelin disruption plays a key role in both the onset and progression of symptoms (Wang et al., 2025), a detailed examination of the brain structures potentially affected by the underlying myelin pathology might provide valuable insights into the clinical course of DEACMP.

Early neuroimaging studies in DEACMP identified characteristic focal lesions, particularly globus pallidus necrosis, alongside nonspecific cortical atrophy (Chang et al., 1992). Subsequently, diffuse white matter damage was recognized as the central pathologic feature of the syndrome (Chang et al., 1992). The observed leukoencephalopathy is characterized by periventricular hyperintensities on T2-weighted magnetic resonance imaging (MRI) and microstructural disruptions in tracts such as the corpus callosum and frontal projections (Fujiwara et al., 2012; Porter, 2002; Chen et al., 2015). Those alterations might contribute not only to persistent cognitive dysfunction, but also (as recent findings suggest) to secondary cortical atrophy, possibly through transneuronal degeneration or deafferentation—indicating a need to revisit cortical involvement in the disease process (Zhang et al., 2023; Zhang et al., 2024).

Recent morphometric studies have reemphasized the role of cortical abnormalities in DEACMP, identifying structural deficits such as reduced cortical thickness or gray matter volume in key prefrontal regions, including the superior and middle frontal gyri (Chen et al., 2013; Wang et al., 2023). Those structural changes, most prominently observed after the onset of the delayed symptoms, have been linked to long-term cognitive outcomes (Chen et al., 2013; Wang et al., 2023). However, the foregoing findings have been derived primarily from cross-sectional studies using conventional morphometric analyses. Although informative, such approaches provide limited insight into the dynamic trajectory of cortical pathology during the subacute recovery phase, when cognitive functioning (including executive functioning) often undergoes rapid change. Notably, no previous study has longitudinally assessed both cortical thickness and intracortical microstructural integrity, including myelin-related changes, within a single individual after DEACMP onset.

Among the neuroimaging techniques used to probe myelin integrity, the T1-weighted to T2-weighted (T1w/T2w) signal intensity ratio has been proposed as a surrogate marker of intracortical myelin content, showing good correspondence with histologic myelin density (Glasser and Van Essen, 2011; Sandrone et al., 2023). That method enables *in vivo* mapping of the intracortical microstructure with relatively high spatial resolution. Importantly, the T1w/T2w ratio is also sensitive to other tissue properties such as neuroinflammation and edema that alter tissue water content. T1w/T2w mapping has been applied in various neurologic conditions, including multiple sclerosis (Cooper et al., 2019), but has yet to be explored in DEACMP. Because DEACMP involves an interaction between demyelination and inflammatory processes (Huang et al., 2020), T1w/T2w mapping offers a promising avenue for capturing dynamic cortical changes that might underlie cognitive and behavioral symptoms.

Here, we present the case of a middle-aged man who developed DEACMP during prophylactic hyperbaric oxygen therapy (HBOT) for acute carbon monoxide poisoning. To our knowledge, this longitudinal investigation is the first to simultaneously evaluate intracortical microstructural alterations (through T1w/T2w myelin mapping) and cortical thickness in the same individual during the early recovery phase. Furthermore, it explores the temporal associations between those cortical metrics and cognitive function over time. By combining morphometric and microstructural assessments, we hoped to provide new insights into the region-specific vulnerabilities and plasticities of the cortex in DEACMP.

## Case description

### Clinical course

#### Acute carbon monoxide poisoning phase (days 0–9)

A 51-year-old man attempted suicide by burning charcoal after severe work-related psychological stress. His carboxyhemoglobin level was 36.2% on day 0. HBOT was initiated the next day (day 1). The patient was then transferred to the psychiatric ward (day 9) after completing five HBOT sessions.

In the psychiatric ward, the Revised Hasegawa Dementia Scale (HDS-R), Mini–Mental State Examination (MMSE), and Frontal Assessment Battery (FAB) were repeatedly administered to track longitudinal change in cognitive function, including executive functioning. For this case report, executive functioning was operationally defined as performance on the FAB. For reference, normal is generally considered to be 21 or greater on the HDS-R, 24 or greater on the MMSE, and 14 or greater on the FAB (Kato et al., 1991; Folstein et al., 1975; Dubois et al., 2000).

#### Lucid interval and onset of DEACMP (days 9–23)

Brain MRI (session 1, day 9) revealed no obvious signal abnormalities, including in the globus pallidus or deep white matter. Bedside cognitive testing on the same day indicated partial recovery from coma, with borderline global cognition (HDS-R/MMSE: 21/24) and preserved executive functioning (FAB: 16). Although global scores improved by day 17 (23/28), the FAB declined to 12, suggesting secondary emergence of executive dysfunction. Electroencephalography (EEG) on the same day revealed irregular slow waves predominantly in the bilateral frontopolar regions.

By day 21, the patient had developed psychomotor slowing, bradyphrenia, and features suggestive of akinetic mutism, with HDS-R/MMSE/FAB scores sharply declining (8/11/7). Repeat electroencephalography demonstrated progression of irregular slow waves to the middle temporal lobes and generalized background slowing (7–9 Hz). MRI session 2 (day 23) revealed widespread high-intensity lesions in the bilateral deep white matter. Cortical atrophy also appeared to have progressed on visual inspection. Those findings supported the diagnosis of DEACMP. Despite the prophylactic HBOT, delayed sequelae nevertheless developed. A cerebrospinal fluid assessment was unremarkable.

#### Progression and recovery phase (days 23–152)

Given the accumulating evidence that therapeutic HBOT can improve cognitive outcomes in established DEACMP (Liao et al., 2021), the patient’s HBOT treatment was continued. By day 47, his cognition had deteriorated markedly (HDS-R/MMSE/FAB: 1/0/0). HBOT was completed on day 56 (30 sessions total, the standard course at our institution). On day 60, MRI session 3 revealed progression of the deep white matter lesions since session 2, and cognitive function remained severely impaired (3/3/3). However, around this time, subtle signs of clinical improvement emerged: facial expressivity became more frequent, flexible, and positive, accompanied by spontaneous eye contact. Electroencephalography on day 67 was already normal. By day 81 (MRI session 4), global cognitive scores had improved (HDS-R/MMSE: 21/20), but executive functioning remained impaired (FAB: 6). On day 115 (MRI session 5), cognitive scores had further improved (23/22/11), with some evident recovery.

**FIGURE 1:**
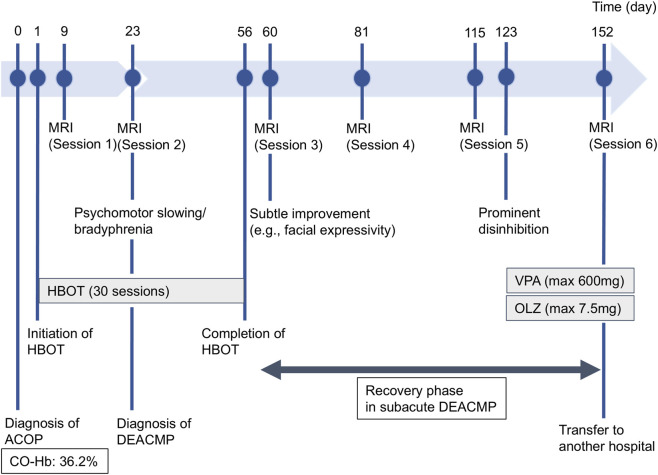
Timeline of the patient’s clinical course. Key events in the patient’s clinical chronology from admission to transfer (days 1–153) are illustrated, including the timing of diagnoses (acute carbon monoxide poisoning and delayed encephalopathy after acute carbon monoxide poisoning [DEACMP]), major therapeutic interventions including hyperbaric oxygen therapy (HBOT) and pharmacotherapy (valproate [VPA] and olanzapine [OLZ]), serial magnetic resonance imaging (MRI) sessions, and the evolution of prominent neuropsychiatric symptoms.

At approximately day 123, the patient developed prominent disinhibition, including excessive demands, logorrhea, and irritability, without disturbance of consciousness. Those symptoms responded well to valproate and olanzapine. On day 140, the patient’s FAB score reached 14, the first nonimpaired level since day 9. The final MRI (session 6, day 152) demonstrated no marked interval change in the deep white matter lesions since MRI session 3. The patient was transferred to another facility on day 153 (Figure 1).

### Follow-up and outcomes

HBOT began on Day 1 to treat acute CO poisoning and prevent DEACMP. Nevertheless, DEACMP developed by Day 23, at which time T2w MRI showed widespread hyperintense lesions in the bilateral deep white matter. HBOT was continued for a total of 30 sessions through Day 56. Executive function (FAB) improved more gradually than global cognition (HDS-R/MMSE). Marked behavioral disinhibition around Day 123 improved with valproate and olanzapine. By Day 140, FAB scores had also reached the lower end of the normal range, along with HDS-R and MMSE, whereas the deep white-matter hyperintensities showed no marked interval change on follow-up MRI. No noteworthy HBOT- or medication-related adverse events were recorded.

### Patient perspective

The patient had limited recall of the early phase of his illness and expressed little concern about his cognitive or behavioral changes, focusing instead on day-to-day matters. He was pleased that he could work hard in his daily rehabilitation and enjoy brief social contacts, and he smiled when staff told him he was getting better.

### Neuroimaging acquisition and processing

High-resolution T1w and T2w magnetic resonance images with a slice thickness of 1 mm were acquired at four time points on a 3T General Electric scanner. T1w/T2w (myelin) maps were derived using the Human Connectome Project minimal preprocessing pipelines during the subacute recovery phase after the onset of DEACMP (MRI sessions 3–6) (Glasser et al., 2013), and analyses emphasized within-subject longitudinal change rather than absolute values. Cortical thickness was estimated using the FreeSurfer (version 6.0.0: Laboratories for Computational Neuroimaging, Charlestown, MA, United States) longitudinal reconstruction processing stream during the same period (Reuter et al., 2012). Detailed acquisition parameters are provided in Supplementary Material S1.

Regions of interest were categorized by functional network, based on the Desikan–Killiany and Glasser cortical parcellations (Desikan et al., 2006; Glasser et al., 2016), and included these networks: (1) frontopolar/dorsolateral prefrontal network, (2) orbitofrontal/ventrolateral network, (3) premotor network, (4) dorsal attention network (DAN), (5) core default mode network (DMN-core), and (6) medial temporal DMN subsystem. For detailed neuroanatomic definitions of each area, see Supplementary Table S1.

## Results

**FIGURE 2:**
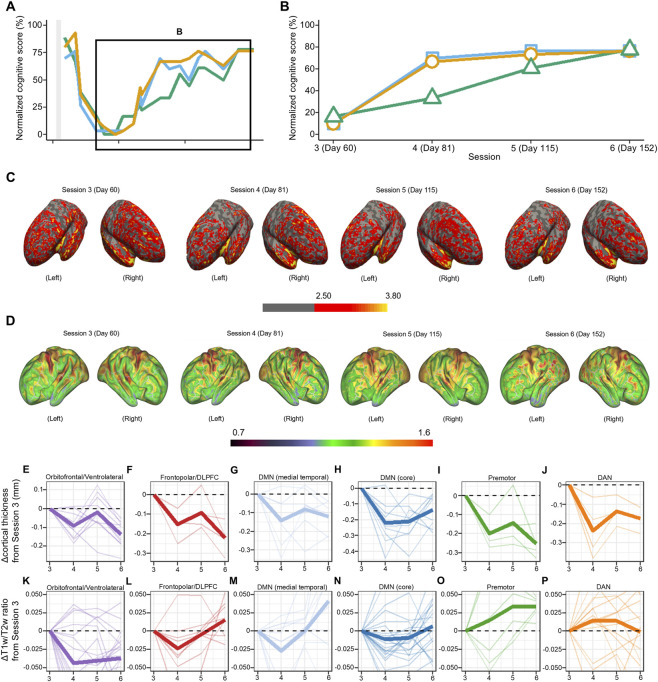
Longitudinal changes in cognitive function, cortical thickness, and T1-weighted/T2-weighted (T1w/T2w) signal intensity ratio during the recovery phase from delayed encephalopathy after acute carbon monoxide poisoning (DEACMP). **(A)** Full time course of normalized (0–100) cognitive scores. The black rectangle, expanded in **(B)**, highlights the recovery phase from magnetic resonance imaging (MRI) session 3 (day 60) to session 6 (day 152). Orange line, Mini–Mental State Examination (MMSE); teal line, Revised Hasegawa Dementia Scale (HDS-R); green line, Frontal Assessment Battery (FAB). Scores were normalized to percentages using (observed score/test-specific maximum) × 100 (maximum scores: MMSE, 30; HDS-R, 30; FAB, 18). For reference, commonly used lower thresholds for normal performance are 70% for HDS-R (21/30), 80% for MMSE (24/30), and 78% for FAB (14/18). **(C)** Longitudinal cortical thickness and **(D)** intracortical myelin maps during recovery from DEACMP. Lateral views of the left and right hemispheres at MRI sessions 3 (day 60), 4 (day 81), 5 (day 115), and 6 (day 152). **(C)** Color scale 2.50–3.80 mm; warmer colors indicate greater cortical thickness (cooler colors indicate cortical thinning). **(D)** Color scale 0.70–1.60; warmer colors indicate higher T1w/T2w ratios, reflecting greater intracortical myelin signal. **(E–J)** Longitudinal change in cortical thickness relative to the MRI session 3 baseline across six functional networks: **(E)** orbitofrontal/ventrolateral, **(F)** frontopolar/dorsolateral prefrontal, **(G)** medial temporal DMN subsystem, **(H)** core default mode network (DMN-core), **(I)** premotor, **(J)** dorsal attention network (DAN). Each bold line denotes the mean change in cortical thickness (in millimeters) across the constituent subregions; the thin lines denote the individual subregions. Positive values indicate thickening relative to MRI session 3; negative values indicate thinning. **(K–P)** Longitudinal changes in the T1w/T2w ratio relative to the MRI session 3 baseline for the same networks. (Panel order as in E–J.) Each bold line denotes the mean change in T1w/T2w (unitless) across the constituent subregions; the thin lines denote individual subregions. Positive values indicate a higher T1w/T2w ratio relative to MRI session 3; negative values indicate a lower ratio.

The patient’s cortical structure was evaluated using brain MRI at four time points during the recovery phase from acute DEACMP, accompanied by repeated cognitive assessments. Quantitative analyses began with MRI session 3 (day 60), defined as the clinical baseline of the recovery phase based on the emergence of subtle cognitive and behavioral improvements (HDS-R/MMSE/FAB: 3/3/3) after the most severe phase of impairment (Figures 2A,B). Figures 2C,D illustrate longitudinal cortical thickness and intracortical myelin maps from MRI session 3 to session 6. To provide a qualitative visualization of the structural change, overlays of coregistered T1w images from Sessions 3 and Session 6 at corresponding axial and coronal levels are shown in Supplementary Figure S1, highlighting ventricular enlargement and sulcal widening consistent with global cortical atrophy.

By MRI session 4 (day 81), a dissociation in cognitive recovery was evident: Although global cognitive scores showed marked improvement (HDS-R/MMSE: 21/20), executive functioning remained severely impaired (FAB: 6). This clinical improvement was observed despite MRI findings of continued cortical thinning (Figures 2E–J) and reduced T1w/T2w ratios in many regions between MRI sessions 3 and 4 (Figures 2K–P). However, during the same period, increases in the T1w/T2w ratio were observed in several functional networks, including the premotor cortex and the DAN (Figures 2O,P).

Between MRI session 4 (day 81) and the final follow-up at session 6 (day 152), a continued decline in cortical thickness was observed in most regions. In contrast, the T1w/T2w ratio increased notably in some frontal and DMN-related regions, paralleling the improvements in the FAB score (to 14 from 6). However, T1w/T2w increases in the orbitofrontal and ventrolateral regions remained limited throughout the observation period, a finding consistent with the clinical emergence of marked disinhibition and emotional lability at approximately day 123 (Figure 1). In that later recovery phase, the improvement in global cognition scores plateaued before reaching full recovery, even as the FAB continued to improve. That cognitive dissociation was mirrored by divergent patterns of microstructural change: Whereas several frontal and DMN-related regions showed sustained T1w/T2w increases, the T1w/T2w ratio within the DAN reversed its initial gains and began to decline (Figure 2P).

## Discussion

This case report presents the first longitudinal investigation into the relationship between cortical pathology—namely, cortical thickness and intracortical microstructure—and clinical recovery in a patient with subacute DEACMP. Our findings revealed that, in this patient, changes in the intracortical microstructure, as measured by the T1w/T2w ratio, appeared to track the clinical course more closely than did cortical thickness.

Despite continued diffuse cortical thinning during the subacute phase, global cognition—including executive functioning—improved, and an early increase in the T1w/T2w ratio was evident across several cortical networks. That divergence might reflect two parallel pathologic processes: one involving irreversible structural loss, and the other representing a potentially reversible microstructural response. The progressive cortical thinning likely resulted from irreversible degeneration triggered by the initial hypoxic–ischemic insult and subsequent neuroinflammatory cascades (Thom et al., 2004; Rose et al., 2017; Beppu, 2014; Huang et al., 2020). In contrast, increases in the T1w/T2w ratio pointed to a reversible microstructural process, such as remyelination or edema resolution (Huang et al., 2020). Because substantial remyelination typically requires months to become evident (Terajima et al., 2008; Franklin and Goldman, 2015), the relatively rapid changes observed in our patient are more plausibly explained by edema resolution, with a possible contribution from nascent remyelination. In this framework, the clearance of excess free water from the cortical tissue would yield a swift increase in the T1w/T2w ratio (Ho et al., 2012). HBOT might have facilitated that normalization by improving tissue oxygenation and attenuating inflammatory cascades (Bin-Alamer et al., 2024).

Notably, the T1w/T2w ratio is not strictly myelin-specific and can also vary with tissue water and iron; accordingly, we interpret the increases as evidence of microstructural rather than purely myelin improvement (Uddin et al., 2018). In addition, progressive cortical thinning can influence the T1w/T2w ratio through partial volume effects at the gray–cerebrospinal fluid interface, so some apparent longitudinal changes—particularly in regions with marked atrophy—may partly reflect geometric rather than purely microstructural factors (Glasser and Van Essen, 2011; Sandrone et al., 2023).

Beyond the evident cross-modal divergence, the T1w/T2w ratio itself showed regional heterogeneity: Increases within key frontal networks—particularly the premotor and dorsolateral prefrontal cortices—were temporally associated with improvements in executive functioning and could be interpreted as early microstructural recovery. In contrast, the T1w/T2w ratios in the orbitofrontal and ventrolateral prefrontal regions remained persistently low, coinciding with the emergence of behavioral disinhibition. Clinically, the marked behavioral disinhibition improved in close temporal proximity to the initiation of valproate and olanzapine, even though T1w/T2w ratios in the orbitofrontal and ventrolateral prefrontal network showed no clear upward trend and cortical thinning progressed over the same interval. This pattern is therefore most consistent with a pharmacologic modulation of behavior rather than cortical microstructural recovery detectable with the present measures. Those contrasting trajectories indicate anatomically heterogeneous cortical change in DEACMP, reflecting region-specific vulnerabilities and capacities for microstructural repair or resilience (Chen et al., 2013). Thus, even as irreversible cortical atrophy—including neuronal loss—progresses, partial and reversible intracortical microstructural improvement might contribute to early clinical recovery in subacute DEACMP.

This report has several limitations. First, being a single case report, its findings might not be generalizable to all patients with DEACMP, a syndrome whose clinical course can be highly heterogeneous. Second, our quantitative imaging analysis commenced at MRI session 3 (day 60), after the clinical nadir had passed, which prevented complete characterization of the entire pathologic trajectory. Third, our analysis focused exclusively on the cortex, and changes in the deep white matter—often considered the primary pathology in DEACMP—were assessed only visually, precluding not only a direct quantitative comparison between gray and white matter recovery, but also an exploration of how primary white matter injury might secondarily affect cortical integrity. Future longitudinal studies simultaneously quantifying both cortical and white matter pathology are needed to fully elucidate the complex interplay between the two compartments. Fourth, our network-level behavioral inferences—especially regarding orbitofrontal contributions to disinhibition—were not directly tested neuropsychologically, because our bedside assessment was limited to the FAB, which, although pragmatic, lacks anatomical specificity (Dubois et al., 2000). Direct verification of these inferences will require a more differentiated battery targeting dorsolateral prefrontal mechanisms (set-shifting/working-memory) (Owen et al., 2005), medial/anterior cingulate functions (response inhibition) (Carter and van Veen, 2007), and orbitofrontal-sensitive behavioral tests (value-based decision-making and probabilistic reversal learning) (Hornak et al., 2004; Tsuchida et al., 2010), together with informant-based disinhibition ratings. Fifth, it is impossible to disentangle the effects of the multiple interventions the patient received, most notably a full course of HBOT, from the natural history of the disease.

In conclusion, this case study suggests that, in subacute DEACMP, clinical recovery might be more closely linked with intracortical microstructural improvement than with ongoing global macrostructural cortical atrophy. T1w/T2w mapping therefore holds promise as a potential biomarker for specific aspects of cortical repair and the corresponding functional networks in DEACMP. Future prospective studies in larger patient cohorts are warranted to validate these preliminary observations and to establish the T1w/T2w ratio as a reliable biomarker for evaluating therapeutic interventions in DEACMP.

## Data Availability

The datasets presented in this article are not readily available because they consist of individual-level clinical and neuroimaging data from a single patient and are subject to patient privacy and institutional regulations. Requests to access the datasets should be directed to the corresponding author.
