# Supplementary material for: Cortical microstructural change linked to clinical recovery in subacute delayed encephalopathy after acute carbon monoxide poisoning: a longitudinal case report
Source: Front Toxicol. 2025 Dec 5;7:1701308. doi: 10.3389/ftox.2025.1701308 (PMC12714658; doi:10.3389/ftox.2025.1701308)
Supplement: Supplementary file 1 [file DataSheet1.docx]

Supplementary Material

Cortical microstructural change linked to clinical recovery in subacute delayed encephalopathy after acute carbon monoxide poisoning: a longitudinal case report

Authors

Takehiro Tamura, M.D., Ph.D., Yuka Fujimoto, M.D., Ph.D.,
Hironobu Nakamura, M.D., Ph.D., Yuki Takahashi, M.D.,
Junya Fujino, M.D., Ph.D., Shunsuke Takagi, M.D., Ph.D.,
Hidehiko Takahashi, M.D., Ph.D., and Genichi Sugihara, M.D., Ph.D.

Contents

1. Methods S1: Magnetic Resonance Imaging Acquisition Parameters
2. Table S1: Functional classification of cortical regions of interest (ROIs) based on the Glasser and Desikan–Killiany atlases
3. Figure S1: Qualitative visualization of brain atrophy between Session 3 and Session 6
4. References

Methods S1: Magnetic Resonance Imaging Acquisition Parameters

During each magnetic resonance imaging session, high-resolution T1-weighted (T1w) and T2-weighted (T2w) brain images were acquired using a 3T General Electric scanner. The T1w sequence used these imaging parameters: repetition time, 600 ms; echo time, 10.1 ms; flip angle, 90 degrees. The T2w sequence used these imaging parameters: repetition time, 2500 ms; echo time, 77.8 ms. For both sequences, the voxel size was 0.47 × 0.47 × 1.0 mm (in-plane resolution 0.47 × 0.47 mm; slice thickness 1.0 mm). The T1w and T2w images were acquired during the same imaging session to facilitate subsequent co-registration and processing.

Table S1: Functional classification of cortical regions of interest (ROIs) based on the Glasser and Desikan–Killiany atlases

| Functional network | Constituent ROIs^a^ | |
| --- | --- | --- |
|  | (Glasser atlas)(Glasser et al., 2016) | (Desikan–Killiany atlas)(Desikan et al., 2006) |
| **Frontopolar/DLPFC** | a9–46v, p9–46v, 46, 8C | rostralmiddlefrontal |
|  |  | frontalpole |
| **Orbitofrontal/ Ventrolateral cortices** | IFJa, a47r, p47r, AVI, FOP1, FOP2, FOP3, FOP4, FOP5 | lateralorbitofrontal |
|  |  | medialorbitofrontal |
|  |  | parsopercularis |
|  |  | parsorbitalis |
|  |  | parstriangularis |
| **Premotor cortex** | 6a, 6r, 6d | caudalmiddlefrontal |
|  |  | precentral |
| **DAN** | 7AL, LIPd, PFt, AIP, 7PC, VIP, IP0 | superiorparietal |
|  |  | supramarginal |
| **DMN-core** | 7m, 23d, 31pv, p32, 10r, 10d, 10v, PGp, PFm, PGi, a32pr | isthmuscingulate |
|  |  | posteriorcingulate |
|  |  | precuneus |
|  |  | rostralanteriorcingulate |
|  |  | superiorfrontal |
| **DMN-medial temporal subsystem** | PHA1, PHA2, PHA3, EC, PeEc, TF | entorhinal |
|  |  | fusiform |
|  |  | inferiortemporal |
|  |  | parahippocampal |

^a^ For all functional systems, the listed ROIs from both the left and right hemispheres were included.

DLPFC, Dorsolateral prefrontal cortex; DAN, Dorsal attention network; DMN, Default mode network.


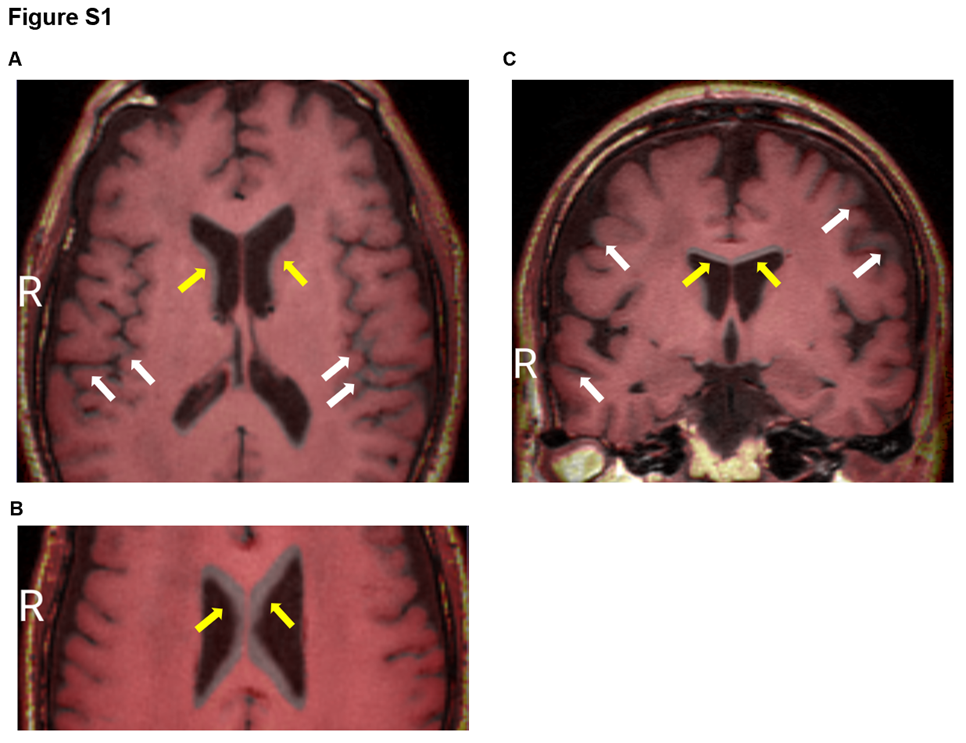
Figure S1: Qualitative visualization of brain atrophy between Session 3 and Session 6

(A, B) Axial and (C) coronal T1-weighted images showing overlays of coregistered scans from Session 3 and Session 6 at corresponding levels. Yellow arrows highlight ventricular enlargement and periventricular atrophy around the frontal horns, and white arrows (A, C) indicate sulcal widening along the frontal/frontoparietal cortices, consistent with global cortical atrophy. Panel B shows an axial close-up at the level of the frontal horns. R indicates the right side of the brain.

References

DESIKAN, R. S., SEGONNE, F., FISCHL, B., QUINN, B. T., DICKERSON, B. C., BLACKER, D., BUCKNER, R. L., DALE, A. M., MAGUIRE, R. P., HYMAN, B. T., ALBERT, M. S. & KILLIANY, R. J. 2006. An automated labeling system for subdividing the human cerebral cortex on MRI scans into gyral based regions of interest. *Neuroimage,* 31**,** 968-80.

GLASSER, M. F., COALSON, T. S., ROBINSON, E. C., HACKER, C. D., HARWELL, J., YACOUB, E., UGURBIL, K., ANDERSSON, J., BECKMANN, C. F., JENKINSON, M., SMITH, S. M. & VAN ESSEN, D. C. 2016. A multi-modal parcellation of human cerebral cortex. *Nature,* 536**,** 171-178.
